# Safety of a dust mite extract in severe allergic asthma

**DOI:** 10.1186/2045-7022-3-S1-P30

**Published:** 2013-05-03

**Authors:** Ruperto González-Pérez, Paloma Poza-Guedes, Inmaculada Sánchez-Machín, Victor Matheu

**Affiliations:** 1 Hospital del Tórax-Ofra, Spain; 2 Hospital del Tórax-Ofra, Allergy, Spain

## Background

The main goal of the current study was to evaluate the clinical tolerance of a modified dust mite subcutaneous extract in a small subset of severe extrinsic asthmatic subjects.

## Methods

We selected 3 adult patients with a confirmed diagnosis of severe persistent allergic asthma (ATS criteria for severe asthma) for at least five years. Mean average topical steroid (inhaled) daily dose was above 1000 mcg of fluticasone propionate or 1600 mcg of budesonide. All patients included in the current trial were clinically relevant sensitized to dust mites (*Dermatophagoides* spp.) as shown by skin prick test and/or specific IgE. Inclusion criteria required no hospital or emergency admissions for the last 2 months with no changes in their daily medication in the last four weeks prior to the administration of immunotherapy. No pre-treatment with systemic steroids and/or antihistaminics were used. Modified standardized specific dust mites immunotherapy extracts were subcutaneously administered according to a validated protocol to achieve a final dose of 100 DDP/ml in a two-week cluster schedule. None of the subjects in both groups have been previously treated with omalizumab. Clinical observation and lung function was strictly monitored in all subjects until the maintenance dose of immunotherapy was reached.

## Results

The three patients could reach the proposed allergen dose according to the immunotherapy schedule in two weeks. No significant adverse reactions were recorded, with no changes in the lung function. Minor immediate local reactions at the injections site were only observed at the maximal allergen dose in 2 patients showing a good response to oral antihistaminics in all cases. No late adverse reactions were present.

## Conclusion

The standardized specific allergen immunotherapy dose was successfully reached in a small group of severe persistent allergic asthmatics. Further studies are needed to evaluate the role of specific immunotherapy in controlled severe allergic asthma.

